# Gender Differences and Associated Factors Influencing Problem Gambling in Adolescents in Sweden: Cross-sectional Investigation

**DOI:** 10.2196/35207

**Published:** 2022-03-17

**Authors:** Emma Claesdotter-Knutsson, Frida André, Maria Fridh, Carl Delfin, Anders Håkansson, Martin Lindström

**Affiliations:** 1 Lund Clinical Research on Externalizing and Developmental Psychopathology (LU-CRED) Child and Adolescent Psychiatry, Section IV, Department of Clinical Sciences, Lund, Faculty of Medicine Lund University Lund Sweden; 2 Clinical Addiction Research Unit, Psychiatry (Lund) Section IV, Department of Clinical Sciences, Lund, Faculty of Medicine Lund University Lund Sweden; 3 Social Medicine and Health Policy Department of Clinical Sciences, Malmo, Faculty of Medicine Lund University Malmö Sweden; 4 Center for Primary Health Care Research (CPF) Malmö Sweden

**Keywords:** gambling, cross-sectional study, adolescents, sleep, alcohol, smoking, pediatrics, parenting, mental health, addiction, children

## Abstract

**Background:**

Although gambling disorder is traditionally considered an adult phenomenon, the behavior usually begins in childhood or adolescence.

**Objective:**

The aim of this study was to explore the frequency of problem gambling among Swedish adolescents and the suspected associated factors.

**Methods:**

This study was based on data collected through a public health survey distributed in 2016 to pupils in ninth grade of primary school and in second grade of secondary school in Sweden. Bayesian binomial regression models, with weakly informative priors, were used to examine whether the frequency of the associated factors differed between those with and without problem gambling.

**Results:**

Approximately 11.7% (469/4002) of the boys in ninth grade of primary school and 13.9% (472/3407) of the boys in second grade of secondary school were classified as problem gamblers. For girls, the corresponding frequencies were 1.2% (48/4167) and 0.7% (27/3634), respectively. The overall response rate was 77% (9143/11,868) among ninth grade pupils and 73.4% (7949/10,832) among second grade pupils, resulting in a total of 17,092 responses. Problem gambling was associated with poor sleep and having tried smoking, alcohol, and other substances among both boys and girls in ninth grade of primary school and boys in second grade of secondary school. Problem gambling among girls in second grade of secondary school was associated with an increased prevalence of having tried smoking and other substances and an increased prevalence of poor sleep.

**Conclusions:**

Using a large representative sample of Swedish adolescents, we found that problem gambling was robustly associated with a substantially increased prevalence of poor sleep and having tried smoking, alcohol, and other substances among both boys and girls in ninth grade of primary school as well as among boys in second grade of secondary school. Our study adds important information for policy makers pointing at vulnerable groups to be considered in their work to prevent problem gambling.

## Introduction

Behavioral or nonsubstance addictions have relatively been formally acknowledged recently [1,2]. The fifth edition of the Diagnostic and Statistical Manual of Mental Disorders includes a new diagnostic category “Substance-Related and Addictive Disorders,” listing not only alcohol and drug abuse but also gambling disorder [1]. Although gambling disorder is traditionally considered an adult phenomenon, the behavior usually begins in childhood or adolescence and more frequently in younger ages among males, in resemblance with both substance use disorders and pathological gaming [2]. Gambling disorder is the most established and most thoroughly investigated behavioral addiction based on a formal diagnosis and founded diagnostic criteria [1]. The research on gambling is rather extensive and includes literature focusing on the Swedish population [3,4], though mainly among adults. The severity of this behavior is currently uncontroversial, as this condition has been associated with negative psychological consequences, including an increased risk of suicide [4-6]. Previous research also demonstrates that gambling disorder shows great comorbidity with various psychiatric conditions such as depression, anxiety disorders, low impulse control, and bipolar disorder as well as alcohol, substance, and nicotine use [2,6,7]. Kessler et al [8] showed that nicotine, alcohol, and drug dependence elevated the odds of pathological gambling. The behavior is traditionally considered mainly an adult problem, but research has shown that problem gamblers debut in gambling at a younger age than nonproblem gamblers [8]. Additionally, previous research concerning comorbidity relies on treatment-seeking samples [4,6,7], and as little is known about gambling in a younger population, this study adds to the knowledge about gambling by addressing early debuting gambling among girls and boys in an ordinary school setting. The psychological health among adolescents is on the decline in the western world, and recent research suggests that this decline is associated with the digital technological development, known as “digital depression” [9,10]. A major study in the United States showed that about 22% of teenagers exhibit multiple symptoms of depression, whereas the lifetime overall US prevalence rate of a full clinical depressive episode is 5%-10% [9,10]. Psychological well-being among adolescents has been reported as poorer among those who spend more hours on electronic communication and in front of screens (eg, social media, gaming, internet, texting) [10].

Autism spectrum disorder (ASD) is an impairing and heterogeneous neurodevelopmental disorder with an early onset and a worldwide prevalence of 1%-3% [11]. This disorder is characterized by social impairments, communication difficulties, altered sensory processing, and repetitive and restricted behaviors [11]. Studies have shown possible social gains for online gamers, decreased feelings of loneliness, increased feelings of connectedness to friends, increased social capital between players, and increased social bridging between players [12]. Based on the design of the games with repetitions and immediate reinforcement, it can be assumed that patients with attention-deficit/hyperactivity disorder (ADHD)/ASD have an increased risk of developing problem gambling [13].

We wanted to explore the frequency of problem gambling among Swedish pupils and examine whether the frequency of the suspected associated factors outlined below differed among those with and without problem gambling. Specifically, using a large sample of Swedish pupils from primary and secondary school, we investigated whether those with and without problem gambling differed in the frequency of (1) often feeling low, (2) often feeling anxious, (3) self-reported ADHD, (4) self-reported ASD, (5) being satisfied with one’s own general health, (6) poor sleep, (7) loneliness, and having tried (8) smoking, (9) alcohol, and (10) other substances.

## Methods

### Participants and Procedures

This study is based on data collected through a public health survey distributed in 2016 to pupils in ninth grade in primary school and in second grade of secondary school. The survey was distributed in all 33 municipalities in Skåne, a region in southern Sweden, with a response rate of 77% (9143/11,868) in ninth grade and 73.4% (7949/10,832) in second grade. Information about gender was missing for 86 respondents, resulting in a total sample size of 17,006. The purpose of the survey was to investigate the current health, way of life, health hazards, and social factors among teenagers and adolescents, and was provided by Region Skåne in cooperation with the municipal association of Skåne. The survey was answered anonymously on computers in classroom settings. Participation was voluntary, all measures were based on self-reports, and all questions were described as optional. In addition to answering questions about problem gambling, respondents were extensively asked about various life circumstances, physical and psychological health factors, and different risk-taking behaviors.

### Measures

#### Problem Gambling

The Lie/Bet questionnaire was used to identify respondents with gambling problems [14,15]. This brief yet diagnostically accurate screening instrument [16] contains only 2 questions (answers: “yes” or “no”): (1) having felt a need to gamble an increasing amount of money in the hopes of winning back what has been lost and (2) lying about the amount of gambling to people of personal importance. Problem gambling was defined as endorsing at least one of these 2 questions.

#### Associated Factors

Based on previous research and clinical experience, we wanted to examine a broad range of suspected associated factors related to overall well-being, mental health, and adverse behaviors. In order to examine the frequency of each factor, new binary variables were created from the available survey questions. Two items based on the Health Behavior in School-aged Children symptom checklist were used to assess respondents’ psychological health, both with separately verified satisfactory test-retest reliability [17]. Respondents rated how often they had “felt low” and “anxious/worried” during the past 6 months on a 5-point scale (about every day, more than once a week, about every week, about every month, rarely or never). Two new binary variables labelled “often feeling low” and “often feeling anxious” were created, where those who answered “about every day” or “more than once a week” were categorized as “yes” and all others as “no.” The survey included several questions on long-term somatic or psychiatric disorders. Respondents were asked whether they had “ADHD or attention-deficit disorder” and “autism/Asperger syndrome.” Two new binary variables labelled ADHD and ASD were created, where those who affirmed ADHD/attention-deficit disorder or ASD were categorized as yes and all others as no. Respondents were asked to rate their general health status on a 5-point scale (very good, rather good, neither good nor poor, rather poor, poor) using the Self-Rated Health instrument [18]. A new binary variable labelled “satisfied with health” was created, with those answering “very good” or “rather good” classified as yes and all others as no. Respondents were asked to rate how many hours a night they usually sleep on weekdays on a 3-point scale (less than 7 hours, 7-9 hours, more than 9 hours). A new binary variable labelled “poor sleep” was created, with those answering “less than 7 hours” classified as yes and all others as no. Respondents were asked to rate on a 4-point scale (have no close friend, have one close friend, have two close friends, have several close friends) whether they presently have a close friend with whom they could talk in confidence about almost any personal matter. A new binary variable labelled “loneliness” was created, with those answering “have no close friend” classified as yes and all others as no. Respondents were asked several questions about smoking, alcohol habits, and illicit substances. Three new binary variables labelled “tried smoking,” “tried alcohol,” and “tried other substances” were created, with affirming of any kind of frequency classified as yes and all other responses as no.

### Statistical Analysis

All statistical analyses were carried out using the R statistical programming language, version 4.0.4 [19], and several functions from the R package tidyverse [20] were used during data processing. Owing to its many advantages over the traditional frequentist approach, including the possibility of making genuine probabilistic statements about estimated parameters [21], we opted for a fully Bayesian approach to statistical analysis. The R package brms [22], which interfaces R with the Stan probabilistic programming language [23], was used to specify Bayesian models. Bayesian binomial regression models with weakly informative priors centered around zero, which should have minimal impact on the obtained estimates while still providing moderate regularization [24], were used to assess whether the frequency of a suspected associated factor was different among those with and without problem gambling. The R package emmeans [25] was used for postprocessing of results. Differences are presented as estimated median absolute percentage differences along with associated odds ratios, both with 95% highest density intervals (HDIs) presented within parentheses. An advantage of the HDI is that, in contrast to a frequentist confidence interval, a 95% HDI actually has a 95% probability of containing the values inside it [26]. Finally, since there is no notion of “statistical significance” in the Bayesian framework, we used the region of practical equivalence in conjunction with the 95% HDI as a decision boundary [26] in order to establish whether an estimated difference between those with and without problem gambling was of practical clinical importance. We considered an estimated difference of 5% (or –5%) as the minimal difference for “practical equivalence,” and if the 95% HDI was not beyond this cutoff, we deemed the results as uncertain in terms of practical importance.

## Results

### Frequency of Problem Gambling Among Swedish School Pupils

Approximately 11.7% (469/4002) of boys in ninth grade of primary school and 13.9% (472/3407) of the boys in second grade of secondary school were classified as problem gamblers. For girls, the corresponding frequencies were 1.2% (48/4167) and 0.7% (27/3634), respectively. Additional details, including the number of valid responses in each group, are presented in Table 1. Overall, 4 factors emerged as robustly more frequent among respondents with problem gambling (although the results varied depending on sex and grade): poor sleep and having tried smoking, alcohol, and other substances.

**Table 1:** Frequency of problem gambling among school pupils in southern Sweden based on data collected in 2016.

School grade and gender	Respondents (N)	Valid responses, n (%)	Problem gambling, n (%)	No problem gambling, n (%)
Boys in ninth grade of primary school	4609	4002 (86.8)	469 (11.7)	3533 (88.3)
Girls in ninth grade of primary school	4497	4167 (92.7)	48 (1.2)	4119 (98.8)
Boys in second grade of secondary school	3945	3407 (86.4)	472 (13.9)	2935 (86.1)
Girls in second grade of secondary school	3955	3634 (91.9)	27 (0.7)	3607 (99.3)

### Problem Gambling and Associated Factors Among Boys in Ninth Grade of Primary School

**Figure 1:**
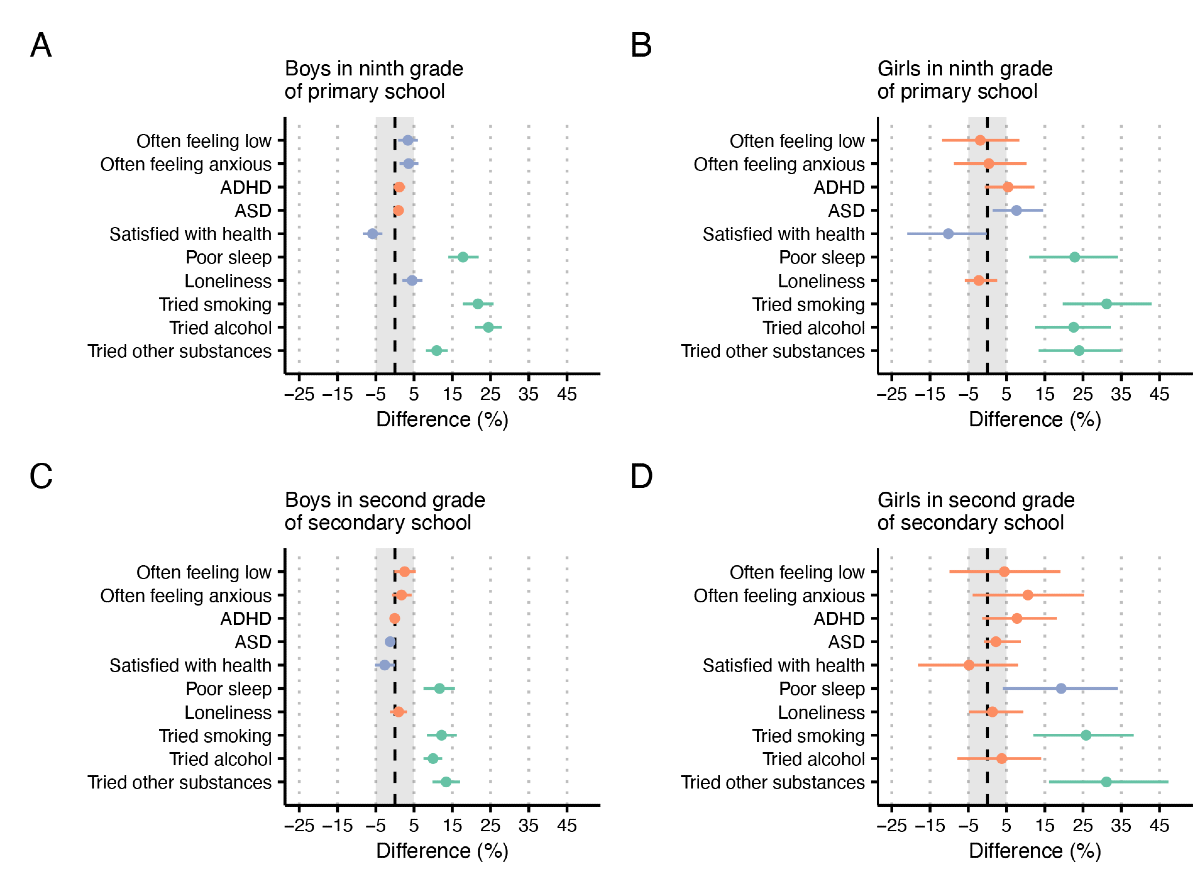
Estimated differences in prevalence of factors that may be associated with problem gambling. Dots represent the posterior median estimate, and bars represent 95% highest density intervals. Shaded regions indicate the region of practical equivalence (ie, a 5% difference in absolute terms). Estimates that with 95% probability are above the region of practical equivalence are shown in green, whereas estimates that with 95% probability are above zero are shown in blue, and estimates that are not, with 95% probability, above zero are shown in orange. Estimates are based on data collected among school pupils in southern Sweden in 2016. ADHD: attention-deficit/hyperactivity disorder; ASD: autism spectrum disorder.

In ninth grade of primary school, 43.3% (202/466) of boys with problem gambling were classified as having poor sleep compared to 25.5% (897/3513) of those without problem gambling, with an estimated difference of 17.8% (14%-21.9%) and a corresponding odds ratio of 2.23 (95% CI 1.86-2.61). Findings were similar for having tried smoking, alcohol, and other substances. Almost half (226/458, 49.3%) of all boys with problem gambling had tried smoking compared to about one-fourth (963/3482, 27.7%) of those without problem gambling, with an estimated difference of 21.7% (17.7%-25.8%) and a corresponding odds ratio of 2.55 (95% CI 2.14-2.98). As for having tried alcohol, this was true for 77.3% (357/462) of those with problem gambling and 52.8% (1857/3514) for those without, with an estimated difference of 24.5% (20.9%-27.9%) and a corresponding odds ratio of 3.04 (95% CI 2.49-3.66). Finally, 15.9% (73/459) of boys with problem gambling had tried other substances compared to 4.9% (172/3480) of those without, with an estimated difference of 10.9% (8.1%-13.8%) and an associated odds ratio of 3.63 (95% CI 2.78-4.56). In addition, there was a robust although smaller group difference for all remaining suspected associated factors except for ADHD and ASD, but the estimated differences were not robustly beyond 5%. Further details are presented in Table 2 and Figure 1A.

**Table 2:** Problem gambling and associated factors among boys in ninth grade of primary school based on data collected in southern Sweden in 2016.

Factors^a^	Boys (n)	Problem gambling, n (%)	No problem gambling, n (%)	Estimated difference in percent (95% highest density interval)^b^	Odds ratio (95% highest density interval)
Often feeling low	3855	52 (11.6)	278 (8.2)	3.4 (0.8 to 6.0)	1.47 (1.10 to 1.87)
Often feeling anxious	3845	46 (10.3)	225 (6.6)	3.6 (1.2 to 6.1)	1.61 (1.18 to 2.08)
Satisfied with health	3919	404 (88.2)	3257 (94.1)	–5.8 (–8.4 to –3.3)	0.47 (0.35 to 0.60)
Attention-deficit/hyperactivity disorder	3852	16 (3.6)	81 (2.4)	1.1 (–0.3 to 2.7)	1.50 (0.85 to 2.22)
Autism spectrum disorder	3853	13 (3)	67 (2)	0.9 (–0.4 to 2.3)	1.49 (0.79 to 2.30)
Poor sleep	3979	202 (43.3)	897 (25.5)	*17.8 (14.0 to 21.9)*	2.23 (1.86 to 2.61)
Loneliness	3964	58 (12.5)	276 (7.9)	4.5 (2.0 to 7.2)	1.66 (1.25 to 2.09)
Tried smoking	3940	226 (49.3)	963 (27.7)	*21.7 (17.7 to 25.8)*	2.55 (2.14 to 2.98)
Tried alcohol	3976	357 (77.3)	1857 (52.8)	*24.5 (20.9 to 27.9)*	3.04 (2.49 to 3.66)
Tried other substances	3939	73 (15.9)	172 (4.9)	*10.9 (8.1 to 13.8)*	3.63 (2.78 to 4.56)

^a^The number of respondents with problem gambling and no problem gambling differ for each factor owing to missing data. The number of respondents for each factor are provided in Table S1 of Multimedia Appendix 1.

^b^Estimated differences and odds ratios with 95% probability that are above the prespecified cutoff for practical equivalence are in italics.

### Problem Gambling and Associated Factors Among Girls in Ninth Grade of Primary School

Girls in ninth grade of primary school classified as problem gamblers had a higher frequency of poor sleep and having tried smoking and other substances than boys in ninth grade, although the frequency of having tried alcohol was similar. Approximately 58% (28/48) of girls with problem gambling were classified as having poor sleep compared to 35.7% (1460/4094) among those without, with an estimated difference of 22.8% (10.9%-34.1%) and an associated odds ratio of 2.54 (95% CI 1.42-3.9). Furthermore, 63% (29/46) of those with problem gambling had tried smoking compared to 31.7% (1306/4082) of those without, resulting in an estimated difference of 31.2% (19.7%-42.9%) and an associated odds ratio of 3.65 (95% CI 1.94-5.71). Almost 4 out of 5, 77% (37/48) of girls with problem gambling had tried alcohol compared to a bit more than half (2252/4102, 54.9%) among girls without, with an estimated difference of 22.5% (12.5%-32.2%) and an associated odds ratio of 2.82 (95% CI 1.35-4.62). Finally, 28% (13/46) of girls with problem gambling had tried other substances compared to 4% (165/4078) of those without, with an estimated difference of 24% (13.4%-35%) and a corresponding odds ratio of 9.25 (95% CI 4.54-14.89). Note, however, that the HDIs presented here are wider than the corresponding HDIs among ninth grade boys owing to the lower number of girls reporting problem gambling. Thus, these estimates are more uncertain. Girls with problem gambling had a higher prevalence of ASD and were less satisfied with their health compared to girls without problem gambling, although these estimates were not robustly beyond 5%. Detailed results are presented in Table 3 and Figure 1B.

**Table 3:** Problem gambling and associated factors among girls in ninth grade of primary school based on data collected in southern Sweden in 2016.

Factors^a^	Girls (n)	Problem gambling, n (%)	No problem gambling, n (%)	Estimated difference in percent (95% highest density interval)^b^	Odds ratio (95% highest density interval)
Often feeling low	4076	10 (22.2)	955 (23.7)	–1.9 (–11.9 to 8.3)	0.9 (0.42 to 1.49)
Often feeling anxious	4071	9 (20)	770 (19.1)	0.4 (–8.8 to 10.2)	1.02 (0.45 to 1.70)
Satisfied with health	4104	35 (74.5)	3449 (85)	–10.2 (–21.0 to –0.2)	0.52 (0.26 to 0.86)
Attention-deficit/hyperactivity disorder	4047	4 (8.9)	118 (2.9)	5.4 (–0.7 to 12.3)	3 (0.69 to 5.92)
Autism spectrum disorder	4032	4 (9.1)	37 (0.9)	7.6 (1.3 to 14.6)	10.09 (2.21 to 20.3)
Poor sleep	4142	28 (58.3)	1460 (35.7)	*22.8 (10.9 to 34.1)*	2.54 (1.42 to 3.90)
Loneliness	4142	2 (4.3)	245 (6)	–2.2 (–5.9 to 2.5)	0.61 (0.04 to 1.46)
Tried smoking	4128	29 (63)	1306 (32)	*31.2 (19.7 to 42.9)*	3.65 (1.94 to 5.71)
Tried alcohol	4150	37 (77.1)	2252 (54.9)	*22.5 (12.5 to 32.2)*	2.82 (1.35 to 4.62)
Tried other substances	4124	13 (28.3)	165 (4)	*24 (13.4 to 35)*	9.25 (4.54 to 14.89)

^a^The number of respondents with problem gambling and no problem gambling differ for each factor owing to missing data. The number of respondents for each factor are provided in Table S2 of Multimedia Appendix 1.

^b^Estimated differences and odds ratios with 95% probability that are above the prespecified cutoff for practical equivalence are in italics.

### Problem Gambling and Associated Factors Among Boys in Second Grade of Secondary School

Notably, differences between those with and without problem gambling were smaller for poor sleep and having tried smoking and alcohol among boys in second grade of secondary school than among boys in ninth grade of primary school, while the difference for having tried other substances stayed more or less the same. Approximately 51.6% (241/467) of boys with problem gambling were classified as having poor sleep compared to 39.9% (1167/2924) of those without, with an estimated difference of 11.7% (7.5%-15.7%) and a corresponding odds ratio of 1.61 (95% CI 1.34-1.87). Furthermore, 66.2% (307/464) of boys with gambling problems had tried smoking, while the same was true for 53.3% (1564/2897) of those without, with an estimated difference of 12.2% (8.4%-16.2%) and a corresponding odds ratio of 1.67 (95% CI 1.38-1.96). The vast majority of boys with (425/464, 91.6%) and without (2380/2915, 81.6%) problem gambling had tried alcohol, with an estimated difference of 10% (7.5%-12.3%) and an associated odds ratio of 2.46 (95% CI 1.78-3.2). Finally, 28.2% (130/461) of those with problem gambling had tried other substances compared to 14.8% (428/2895) of those without, with an estimated difference of 13.4% (9.8%-17%) and an associated odds ratio of 2.26 (95% CI 1.85-2.71). Detailed results are presented in Table 4 and Figure 1C.

**Table 4:** Problem gambling and associated factors among boys in second grade of secondary school based on data collected in southern Sweden in 2016.

Factors^a^	Boys (n)	Problem gambling, n (%)	No problem gambling, n (%)	Estimated difference in percent (95% highest density interval)^b^	Odds ratio (95% highest density interval)
Often feeling low	3308	63 (13.8)	318 (11.2)	2.5 (0.1 to 5.5)	1.27 (0.97 to 1.59)
Often feeling anxious	3313	50 (11)	263 (9.2)	1.7 (–0.8 to 4.4)	1.21 (0.9 to 1.55)
Satisfied with health	3351	406 (88.3)	2630 (91)	–2.7 (–5.2 to 0.0)	0.75 (0.56 to 0.95)
Attention-deficit/hyperactivity disorder	3309	12 (2.7)	76 (2.7)	–0.1 (–1.3 to 1.3)	0.98 (0.49 to 1.5)
Autism spectrum disorder	3311	7 (1.5)	78 (2.7)	–1.2 (–2.2 to –0.1)	0.54 (0.22 to 0.92)
Poor sleep	3391	241 (51.6)	1167 (39.9)	*11.7 (7.5 to 15.7)*	1.61 (1.34 to 1.87)
Loneliness	3381	39 (8.3)	212 (7.3)	1 (–1.3 to 3.2)	1.15 (0.81 to 1.5)
Tried smoking	3361	307 (66.2)	1564 (54)	*12.2 (8.4 to 16.2)*	1.67 (1.38 to 1.96)
Tried alcohol	3379	425 (91.6)	2380 (81.6)	*10 (7.5 to 12.3)*	2.46 (1.78 to 3.2)
Tried other substances	3356	130 (28.2)	428 (14.8)	*13.4 (9.8 to 17.0)*	2.26 (1.85 to 2.71)

^a^The number of respondents with problem gambling and no problem gambling differ for each factor owing to missing data. The number of respondents for each factor are provided in Table S3 of Multimedia Appendix 1.

^b^Estimated differences and odds ratios with 95% probability that are above the prespecified cutoff for practical equivalence are in italics.

### Problem Gambling and Associated Factors Among Girls in Second Grade of Secondary School

Again, owing to the low number of girls with problem gambling in second grade of secondary school, several estimates were uncertain. For instance, while 63% (17/27) of girls with problem gambling and 43.7% (1578/3590) without were classified as having poor sleep, the estimated difference of 19.3% (4%-34%) was not, with 95% probability, above the prespecified cutoff for practical equivalence (although the estimated difference was, with 95% probability, still above zero). As for having tried smoking, this was affirmed by 77% (20/26) of girls with and 51.8% (1857/3585) of girls without problem gambling, with an estimated difference of 25.8% (12%-38.3%) and an associated odds ratio of 3.23 (95% CI 1.09-6.28). Notably, the difference in having tried alcohol was negligible, while 42% (11/26) of girls with and 10.9% (393/3574) of girls without problem gambling had tried other substances, with an estimated difference of 31.1% (16.1%-47.3%) and an associated odds ratio of 5.9 (95% CI 2.5-10.36). Detailed results are presented in Table 5 and Figure 1D.

**Table 5:** Problem gambling and associated factors among girls in second grade of secondary school based on data collected in southern Sweden in 2016.

Factors^a^	Girls (n)	Problem gambling, n (%)	No problem gambling, n (%)	Estimated difference in percent (95% highest density interval)^b^	Odds ratio (95% highest density interval)
Often feeling low	3579	9 (33.3)	1011 (28.5)	4.4 (–9.9 to 19.1)	1.23 (0.49 to 2.15)
Often feeling anxious	3585	9 (33.3)	795 (22.3)	10.6 (–3.9 to 25.3)	1.71 (0.66 to 2.96)
Satisfied with health	3570	20 (76.9)	2918 (82.3)	–4.8 (–18.2 to 8.1)	0.74 (0.26 to 1.46)
Attention-deficit/hyperactivity disorder	3560	3 (12)	118 (3.3)	7.7 (–1.4 to 18.1)	3.61 (0.48 to 7.9)
Autism spectrum disorder	3560	1 (4)	28 (0.8)	2.2 (–1.0 to 8.7)	3.94 (0 to 13.63)
Poor sleep	3617	17 (63)	1578 (44)	19.3 (4 to 34)	2.19 (0.88 to 3.84)
Loneliness	3629	2 (7.4)	186 (5.2)	1.3 (–4.8 to 9.4)	1.27 (0.05 to 3.09)
Tried smoking	3611	20 (76.9)	1857 (51.8)	*25.8 (12 to 38.3)*	3.23 (1.09 to 6.28)
Tried alcohol	3620	22 (84.6)	2935 (81.7)	3.8 (–7.8 to 14)	1.32 (0.38 to 2.96)
Tried other substances	3600	11 (42.3)	393 (11)	*31.1 (16.1 to 47.3)*	5.9 (2.5 to 10.36)

^a^The number of respondents with problem gambling and no problem gambling differ for each factor owing to missing data. The number of respondents for each factor are provided in Table S4 of Multimedia Appendix 1.

^b^Estimated differences and odds ratios with 95% probability that are above the prespecified cutoff for practical equivalence are in italics.

## Discussion

### Principal Findings

Given the increasing interest in behavioral addictions and alarming reports on consequences of screen time and adolescents increasing psychological complaints [2,9,10], this study aimed to describe problem gambling and suspected associated factors within a population of Swedish pupils in an ordinary school setting, targeting adolescents in ninth grade of primary school and in second grade of secondary school. Our study adds to the knowledge of pathological gambling by investigating male and female characteristics. The interest in behavioral addiction is increasing, but there are still gaps to be filled. Gambling addiction is the most established and researched behavioral addiction, but the phenomenon is mainly investigated among adults or within populations of care-seeking gamblers [4,6,7]. Games with or without money constitute adjacent phenomena in the sense that monetary elements such as so-called loot boxes are common in computer games or through more computer-game-like virtual environments where games about money take place. One possibility for affirmation of gambling among adolescents younger than 18 years of age could be that the participants meant games containing such monetary elements when endorsing items on gambling in the questionnaire. The difference in the frequency between girls with and without problem gambling was notably larger than the difference between boys with and without problem gambling for several variables. For instance, the estimated difference in having tried other substances was 11% among boys in ninth grade and 24%, more than double, among girls in ninth grade (see Tables 2 and 3). This might be explained by the fact that girls with problem gambling are fewer but exhibit more severe psychiatric health problems [27,28].

Behavioral and substance addictions have previously been reported as robustly related [2]. Correspondingly, we observed that both male and female problem gamblers in ninth grade displayed a disproportionately high prevalence of having tried cigarettes, alcohol, and illicit drugs. The overrepresentation was seen among male and female problem gamblers in second grade of secondary school, with the exception of the female experience of alcohol. This is well in line with previous research showing that male gamblers drink more and female gamblers less [29]. Owing to legal regulations on gambling, most of the studies were conducted on adult populations, but several studies—some as early as those in 1998 [30]—showed that adolescent gamblers were more likely to drink alcohol, smoke tobacco, and take drugs compared to nongamblers [31]. Theories regarding the relationship between gambling and experience of cigarettes, alcohol, and illicit drugs have been suggested to be excitement-seeking and risk-taking personalities having similar social, environmental, neurobiological, and genetic features [31-34]. Petruzelka et al [35] suggest that the socioeconomic status plays an important role in this bad marriage. Díaz and Pérez [34] found that tobacco and alcohol users are more likely to gamble and spend more on gambling products. Further research is needed to increase the understanding of the causality.

ASD is an impairing and heterogeneous neurodevelopmental disorder with an early onset, which affects 1%-3% of the population [11]. ASD is characterized by social impairments, communication difficulties, altered sensory processing, and repetitive and restricted behaviors [11]. Studies have shown the following possible social gains for online gamers: decreased feelings of loneliness, increased feelings of connectedness to friends, increased social capital between players, and increased social bridging between players [12]. Based on previous research [36,37], we had expected a higher prevalence of gambling in the ASD group but we only found such a relationship among girls in ninth grade. This group had a higher prevalence of ASD and were less satisfied with their health compared to girls without problem gambling. In our study, the total number of participants with ASD was too small (n=20) to draw any conclusions. There is a notable comorbidity between ADHD and ASD [38] and as we only logged the main diagnosis, there might be a possibility that there are some participants with ASD among participants with ADHD and vice versa. Looking at neurodevelopmental disorders from the ESSENCE (Early Symptomatic Syndromes Eliciting Neurodevelopmental Clinical Examinations) perspective, we could have created a group consisting of participants with ASD plus ADHD to obtain a more realistic picture [39]. ASD does not belong to the most frequent conditions investigated in the relationship to a problem or addictive gambling and even less to a potential gender discrepancy. Our results warrant a more thorough investigation of the potential link between neuropsychiatric conditions and problem gambling among female adolescents.

Previous research reports on a relationship between problem gambling and both bad sleeping habits and sleeping difficulties [40]. The association between screen time and sleeping difficulties is established and highly clinically relevant since previous research describes how insufficient sleep is associated with both mental health problems and poor academic performance [41,42]. Concordantly, respondents in our material with problem gambling showed a positive association with <7 hours of sleep per night among female and male ninth graders and among male second graders. Girls with problem gambling showed the same tendency, and it cannot be ruled out that an association would have been seen in a larger data material.

### Strengths and Limitations

Among the strengths of this study are the large, representative sample size, the fact that the study is population-based, and the use of a Bayesian statistical approach, which allowed us to make genuine probabilistic statements about our obtained estimates. However, notwithstanding the strengths of a fully Bayesian approach, the number of girls with problem gambling was relatively small (n=75), and thus, the number of girls with both problem gambling and the presence of an associated factor was even smaller. As indicated by the wider HDIs, our findings pertaining to girls are therefore less robust than those pertaining to boys.

This study has several limitations. First, all measures used were based on self-report, which entails a risk for recall bias that could influence our findings. This study also shows considerable strengths. The survey was population-based and included a large number of individuals and a relatively high response rate, which reduces the risk of selection bias. The only question regarding sleep was “How many hours do you sleep during a weekday?” with 3 fixed alternatives “less than 7 hours,” “7-9 hours,” and “more than 9 hours.” This item might have specified whether the sleep was continuous or intermittent, and a continuous scale for the numbers of hours of sleep during a normal weekday might have been included. However, the sleep item is part of a general public health questionnaire for adolescents in schools in southern Sweden with the aim of reporting the general public health status of this group regarding school, family, demographic, socioeconomic, social capital, social cohesion, social support, social participation/social networks, bullying (physical and cyber bullying), health behavior, somatic health status, psychological health status, exposure to crime, sex habits, and beliefs about the future in a comprehensive and reasonably short questionnaire. Furthermore, to the best of our knowledge, this is the first study to highlight problem gambling and associated factors among Swedish teenagers.

### Conclusion

Using a large representative sample of Swedish adolescents, we found that problem gambling was robustly associated with a substantially increased prevalence of poor sleep and having tried smoking, alcohol, and other substances among both boys and girls in ninth grade of primary school as well as among boys in second grade of secondary school. Owing to the small number of girls with problem gambling in second grade of secondary school, our estimates were less certain, but problem gambling was nevertheless robustly associated with a substantially increased prevalence of having tried smoking and having tried other substances, as well as (less robustly) with an increased prevalence of poor sleep. Furthermore, teenagers with ASD should possible be considered as more likely to engage in problem gambling, specifically girls. Important and clinically relevant questions have been revealed for future studies to answer. Our study adds important information for policy makers pointing at vulnerable groups to be considered in their work to prevent problem gambling.

## Appendix

Multimedia Appendix 1 Supplementary data for tables.

## Abbreviations

ADHD: attention-deficit/hyperactivity disorder
ASD: autism spectrum disorder
ESSENCE: Early Symptomatic Syndromes Eliciting Neurodevelopmental Clinical Examinations
HDI: highest density interval
