# Supplementary material for: Gender Differences and Associated Factors Influencing Problem Gambling in Adolescents in Sweden: Cross-sectional Investigation
Source: JMIR Pediatr Parent. 2022 Mar 17;5(1):e35207. doi: 10.2196/35207 (PMC8972113; doi:10.2196/35207)
Supplement: Multimedia Appendix 1 [file pediatrics_v5i1e35207_app1.docx]

**Table S1.** Data on problem gambling and associated factors among boys in the ninth grade of primary school.

| Factors | Boys (N) | Problem gambling (n) | No problem gambling (n) |
| --- | --- | --- | --- |
| Often feeling low | 3855 | 448 | 3407 |
| Often feeling anxious | 3845 | 448 | 3397 |
| Satisfied with health | 3919 | 458 | 3461 |
| Attention-deficit/hyperactivity disorder | 3852 | 447 | 3405 |
| Autism spectrum disorder | 3853 | 440 | 3413 |
| Poor sleep | 3979 | 466 | 3513 |
| Loneliness | 3964 | 465 | 3499 |
| Tried smoking | 3940 | 458 | 3482 |
| Tried alcohol | 3976 | 462 | 3514 |
| Tried other substances | 3939 | 459 | 3480 |

**Table S2.** Data on problem gambling and associated factors among girls in the ninth grade of primary school.

| Factors | Girls (N) | Problem gambling (n) | No problem gambling (n) |
| --- | --- | --- | --- |
| Often feeling low | 4076 | 45 | 4031 |
| Often feeling anxious | 4071 | 45 | 4026 |
| Satisfied with health | 4104 | 47 | 4057 |
| Attention-deficit/hyperactivity disorder | 4047 | 45 | 4002 |
| Autism spectrum disorder | 4032 | 44 | 3988 |
| Poor sleep | 4142 | 48 | 4094 |
| Loneliness | 4142 | 47 | 4095 |
| Tried smoking | 4128 | 46 | 4082 |
| Tried alcohol | 4150 | 48 | 4102 |
| Tried other substances | 4124 | 46 | 4078 |

**Table S3.** Data on problem gambling and associated factors among boys in the second grade of secondary school.

| Factors | Boys (N) | Problem gambling (n) | No problem gambling (n) |
| --- | --- | --- | --- |
| Often feeling low | 3308 | 458 | 2850 |
| Often feeling anxious | 3313 | 456 | 2857 |
| Satisfied with health | 3351 | 460 | 2891 |
| Attention-deficit/hyperactivity disorder | 3309 | 452 | 2857 |
| Autism spectrum disorder | 3311 | 455 | 2856 |
| Poor sleep | 3391 | 467 | 2924 |
| Loneliness | 3381 | 470 | 2911 |
| Tried smoking | 3361 | 464 | 2897 |
| Tried alcohol | 3379 | 464 | 2915 |
| Tried other substances | 3356 | 461 | 2895 |

**Table S4.** Data on problem gambling and associated factors among girls in the second grade of secondary school.

| Factors | Girls (N) | Problem gambling (n) | No problem gambling (n) |
| --- | --- | --- | --- |
| Often feeling low | 3579 | 27 | 3552 |
| Often feeling anxious | 3585 | 27 | 3558 |
| Satisfied with health | 3570 | 26 | 3544 |
| Attention-deficit/hyperactivity disorder | 3560 | 25 | 3535 |
| Autism spectrum disorder | 3560 | 25 | 3535 |
| Poor sleep | 3617 | 27 | 3590 |
| Loneliness | 3629 | 27 | 3602 |
| Tried smoking | 3611 | 26 | 3585 |
| Tried alcohol | 3620 | 26 | 3594 |
| Tried other substances | 3600 | 26 | 3574 |
